# Quantifying the Costs to Different Funders over Five-Years for Women Diagnosed with Breast Cancer in Queensland, Australia: A Data Linkage Study

**DOI:** 10.3390/ijerph182412918

**Published:** 2021-12-08

**Authors:** Daniel Lindsay, Emily Callander

**Affiliations:** 1 School of Public Health, The University of Queensland, Brisbane 4006, Australia; 2 School of Public Health and Preventative Medicine, Monash University, Melbourne 3004, Australia; Emily.callander@monash.edu

**Keywords:** breast cancer, cost, hospital, emergency department, Medicare, pharmaceutical, out of pocket, long term

## Abstract

Individuals diagnosed with breast cancer have the highest rates of survival among all cancer types. Due to high survival, the costs of breast cancer to different healthcare funders are of interest. This study aimed to describe the cost to public hospital and private health funders and individuals due to hospital and emergency department (ED) admissions, as well Medicare items and pharmaceuticals over five years for Queensland women with breast cancer. We used a linked administrative dataset, CancerCostMod, limited to Queensland female breast cancer diagnoses between July 2011 and June 2013 aged 18 years or over who survived for 5 years (n = 5383). Each record was linked to Queensland Health Admitted Patient Data Collection, Emergency Department Information Systems, Medicare Benefits Schedule, and Pharmaceutical Benefits Scheme records between July 2011 and June 2018. Total costs for different healthcare funders as a result of breast cancer diagnoses were reported, with high costs and service use identified in the first six months following a breast cancer diagnosis. After the first six months post-diagnosis, the financial burdens incurred by different healthcare funders for breast cancer diagnoses in Queensland remain steady over a long period. Recommendations for reducing long term costs are discussed.

## 1. Introduction

Breast cancer is currently the most diagnosed cancer in women, both in Australia and worldwide, and incidence rates in this population are increasing [1,2]. Within Australia, estimated five-year survival rates for breast cancer in women is 92% [3], highlighting the low mortality rates for this disease resulting from significant improvements in breast cancer screening and treatment options. While these high survival rates are positive for those diagnosed with breast cancer, the increased costs of improved treatment options can contribute to major financial burden over time for healthcare systems and the individual due to a breast cancer diagnosis [4,5]. 

In the Australian universal healthcare scheme, public patients within public hospitals are treated free-of-charge, with most of the funding for the public healthcare sector provided by federal and state governments [6]. In contrast, Australian governments provide less funding for privately funded healthcare, with funding mainly coming from other sources, such as private health insurance (PHI) and individuals [6]. Australia has various incentive schemes to encourage individuals to seek care in private hospitals and obtain private health insurance, potentially reducing the strain on public hospitals [7,8]. 

Outside of public hospitals, healthcare is partially funded by the federal government through the Medicare Benefits Schedule (MBS), which provides free public hospital services and free or subsidized primary health care outside of hospitals where individuals may incur a co-payment from their own pockets. The Australian Federal government also provides funding for prescription pharmaceuticals through the Pharmaceutical Benefits Scheme (PBS), with patients also paying a small co-payment when needed. Identifying costs for different funders of healthcare is important when there are several funding sources (as is the case in Australia), as it enables the identification of who pays for healthcare, and whether costs are shifted between funders [9]. 

Various studies have reported the high costs to health system funders associated with a breast cancer diagnosis. For example, Australian health system expenditure on breast cancer was estimated at AU$1056 million in 2015–2016, making it the cancer type with the highest total government expenditure amongst all cancers [10]. Studies from the US have also shown the high financial burdens for breast cancer patients, with one study indicating that cancer patients cost the US healthcare system, on average, approximately four times more than patients without cancer [11], while another found that the total estimated cost of cancer care for privately insured adults was US$156.2 billion [12]. Those diagnosed with breast cancer were found to be among the highest users of medical services, incurring high costs to the healthcare system [12]. Another Australian study focused on direct health system (government funded) costs of cancer consisting of hospital admissions, ED presentations, MBS and PBS items for cancer patients [13]. By comparing the costs incurred by cancer patients to matched controls, this study reported an estimated excess cost of AU$825 million over four to five years post breast cancer diagnosis, with almost three quarters (72%) of these costs occurring in the first-year post diagnosis [13]. As this study focused solely on the healthcare costs to the government, costs to the individual due to a cancer diagnosis were not explored. While some studies have examined the number of ED presentations for breast cancer [14], the costs associated with such visits require further research.

Costs to the individual due to a breast cancer diagnosis have also shown to be high [15,16]. A study by Gordon et al. found that the median out-of-pocket costs for women with breast cancer was AU$4192 in the first two years post-diagnosis [16]. Another study indicated that average co-payments for MBS items in the first three years following a breast cancer diagnosis was AU$1400 (SD = $1946), while average co-payments for pharmaceuticals was $AU974 (SD = $707) in the same period [17]. While providing an indication of the out-of-pocket costs for breast cancer for Australian women, the long-term out-of-pocket costs (costs incurred over five or more years) resulting from a breast cancer diagnosis have yet not been fully explored. These long-term costs are important to determine, as international research suggests that long-term medical costs (four years post-diagnosis) can contribute to significant financial debt for approximately 10% of individuals diagnosed with breast cancer [18].

With the high survival rates of breast cancer, quantifying the costs to different funders resulting from a breast cancer diagnosis over a long period is important. As people are living longer with a breast cancer diagnosis, the costs relating to healthcare for this disease occur over a longer time, placing a significant financial burden on both healthcare systems and individuals. Therefore, the current study aimed to quantify the costs to different funders (public hospital funders, private health funders and individuals) over five years for female breast cancer diagnoses in Queensland, Australia. This is one of the first studies of its kind to quantify the costs of breast cancer diagnoses to different healthcare funders over an extended period and provides a greater understanding of when costs arise to both the healthcare system and individuals through the cancer journey. By using a linked administrative dataset for this purpose, this study can provide a comprehensive overview of the healthcare being accessed by the population of interest and their associated costs. Further, through the hospital coding system used in this dataset, we can estimate the costs of hospital admissions that are related to cancer admissions and those that are for other health issues. This provides a more detailed understanding of the costs being incurred by different healthcare funders when dealing with breast cancer patients.

The remainder of this paper presents the methodology for this study, including an overview of the unique dataset used for data analysis. We then present the costs to different healthcare funders incurred over five years post breast cancer diagnosis. Implications of these findings for the Australian healthcare system are then discussed.

## 2. Materials and Methods

### 2.1. Study Population

The sample used in this study was taken from the CancerCostMod dataset [19,20] which contains records of all cancer diagnoses (excluding non-melanoma skin cancer) in Queensland, Australia between 1 July 2011 and 30 June 2015, as recorded by the Queensland Cancer Registry (QCR) (n = 106,571 patients). Each person’s QCR record is linked to their records in the Queensland Health Admitted Patient Data Collection (QHAPDC), Queensland Health Emergency Department Information Systems (EDIS), MBS and PBS from 1 July 2011 to 30 June 2018. For this study, data was limited to females who were 18 years of age or older when diagnosed with breast cancer (ICD-O C50) between 1 July 2011 to 30 June 2013 to ensure five years of follow-up data (n = 5383). Only individuals who survived an entire five-year period were included in the sample.

The QCR database includes sociodemographic characteristics at time of diagnosis, allowing for the creation of measures for socioeconomic disadvantage, remoteness, and breast cancer stage [19,20,21,22]. The MBS and PBS datasets include information on all MBS services and PBS prescriptions that individuals accessed within the target timeframe. Within the MBS and PBS datasets, information about the date of service/prescription, item code, full charge, government rebate (if applicable), and patient co-payment is reported.

### 2.2. Assigning Costs

The cost of each public hospital admitted episode of care was attributed to the Australian Refined Diagnostic-Related Group (AR-DRG) using the cost as reported by the National Hospital Data Collection (NHCDC) report for the relevant year [23]. Adjustments for certain patient demographics were made to costings to account for variations in costs of delivering healthcare to some individuals [24]. Each ED presentation was coded to the ED classification system Urgency Related Group (URG) using the triage category, discharge destination and primary reason for ED attendance (ICD-10-AM). The cost attributed to each URG for ED presentations was assigned using the mean cost per presentation as reported by the NHCDC Report for the relevant year [23]. Costs to private health funders were created by attributing each AR-DRG for private hospital admissions for the relevant year using the mean charge per separation reported by the Private Hospital Data Bureau (PHDB) Annual Reports [25].

Public and private hospital admissions and their respective costs are reported separately in all analyses. All hospital admissions were also categorized into breast-cancer related and non-cancer related admissions based on the Major Diagnostic Category (MDC) assigned to each DRG record. All MDC values of 9 (diseases of the skin, subcutaneous tissue, and breast) and 17 (myeloproliferative disorders and other neoplasms) were categorized as breast-cancer related admissions, while all other MDC values were categorized as non-cancer related admissions.

Costs to individuals due to a breast cancer diagnosis were created based on the reported patient co-payments for each item/prescription within the MBS and PBS datasets. To limit to those admissions potentially related to treatment for a breast cancer diagnosis, only hospital and ED admissions, and MBS and PBS items/prescriptions occurring after diagnosis were used in analyses. All costs are reported in Australian dollars adjusted to the 2020 calendar year [26].

### 2.3. Statistical Analysis

Descriptive analyses identified the demographic characteristics of the sample. The costs to public and private funders, and individuals, were calculated for each month for each record from the date of diagnosis (t = 0) to 60 months post-diagnosis. If an individual had no health services for the month, the cost was recorded as $0. Costs were then aggregated into six-month time periods from time of diagnosis to 60 months post diagnosis to quantify the costs within each 6-month period. Mean cost per person was calculated by dividing the total costs for each period by the number of persons within the sample. All analyses were undertaken using SAS V9.4 (SAS Institute Inc., Cary, NC, USA).

## 3. Results

Between 1 July 2011 and 30 June 2013, 5383 women aged 18 years or older were diagnosed with breast cancer in Queensland and survived for a period of five or more years. Demographic characteristics at diagnosis for women diagnosed with breast cancer are shown in Table 1. The mean age at diagnosis was 59.4 (SD: 12.4 years).

The total cost of public and private hospital admissions over five years post-diagnosis was $67.4 million and $82.5 million, respectively. See Table 2 for an overview of mean and total costs to different healthcare providers. Cancer-related hospital admissions accounted for $18 million (27%) of total costs to public health funders from public hospital admissions and $36.4 million (44%) of total costs to private health insurers for private hospital admissions. The average cost per person for public hospital admissions over five years was $12,523 (SD = $29,726), while the average cost per person for private hospital admissions over five years was $15,327 (SD = $29,538) (Table 2).

Of the 14,179 total public hospital admissions (mean per person = 2.6, SD = 6.3), 5208 (37%) were cancer-related admissions (mean per person = 1, SD = 4) and 8971 (63%) were non-cancer admissions (mean per person = 1.7, SD = 4). Of the 39,781 total private hospital admissions (mean per person = 7.4, SD = 13.8), 22,945 (58%) were cancer-related admissions (mean per person = 4.3, SD = 9.3) and 16,836 (42%) were non-cancer admissions (mean per person = 3.1, SD = 7.1).

**Figure 1:**
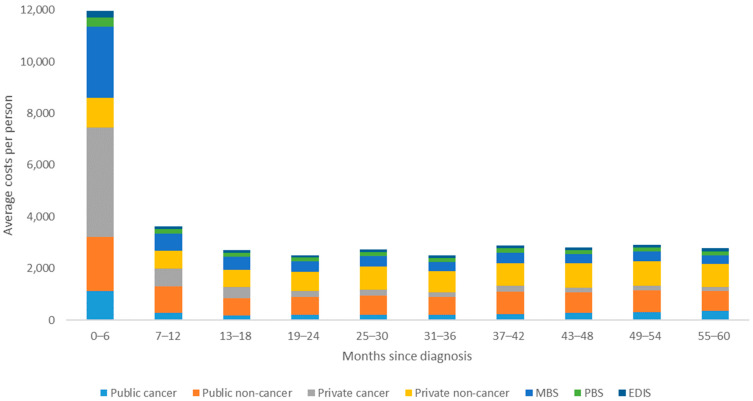
Average cost per person to different health care funders over five years for females diagnosed with breast cancer in Queensland, Australia between 1 July 2011 and 31 June 2013.

**Figure 2:**
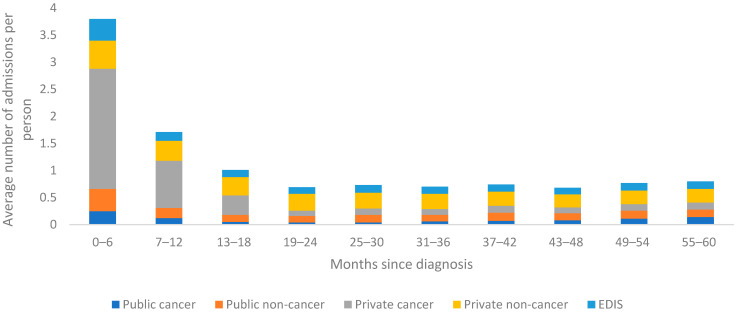
Average number of admissions per person over five years for females diagnosed with breast cancer in Queensland, Australia between 1 July 2011 and 31 June 2013.

Across both public and private hospital admissions, total and average costs and admissions were greatest in the first six-months following diagnosis (see Figure 1 and Figure 2). Within this time frame, cancer-related admissions accounted for only 35% of total costs to public health funders, compared to 79% for cancer-related admissions in private hospitals. After this initial six-month period, the proportion of total costs for cancer-related private hospital admissions becomes lower than the proportion relating to non-cancer related private hospital admissions for the remaining five-year period. Within public hospital admissions, the cost and number of cancer-related admissions remains relatively low in comparison to non-cancer related admissions across the entire five-year period (see Figure 1 and Figure 2).

The total cost to public health funders from ED presentations was $6.1 million over the five-year period, with an average per person of $1132 (SD = $2306) (Table 2). There was a total of 8701 ED presentations for breast cancer patients over the five-year period, with a mean of 1.6 (SD = 3.25) per person. Similar to hospital admissions, individuals reported the highest total and average costs and ED presentations during the first 6 months post-diagnosis. For the remaining time, ED admissions and costs remained relatively similar (see Figure 1 and Figure 2).

**Figure 3:**
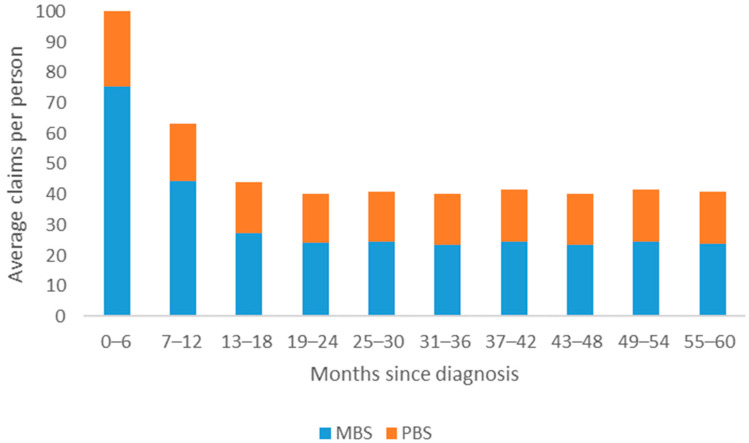
Average number of MBS and PBS items claimed per person over five years for females diagnosed with breast cancer in Queensland, Australia.

The total MBS and PBS costs within this study were $35.4 million and $9.9 million, respectively. There was a total of 1,697,459 MBS items claimed by breast cancer patients over five years, an average of 315 (SD = 187) claims per person. There was a total of 972,649 PBS pharmaceuticals claimed by breast cancer patients over five years, an average of 181 (SD = 145) claims per person. The highest average and total cost and number of items accessed for both MBS and PBS were highest during the first six months post-diagnosis (see Figure 1 and Figure 3). For the remaining time, MBS and PBS items claimed and average costs remained relatively similar. On average, individuals within this sample paid $1836 (SD = $1489) out of their own pocket for pharmaceuticals and $6578 (SD = $7248) on healthcare partially covered by Medicare over the course of five years (Table 2).

## 4. Discussion

This study used linked administrative data to quantify the costs over 5 years to different funders relating to a diagnosis of female breast cancer in Queensland, Australia. The total cost over five years to public health funders was approximately $73.5 million ($67.4 million from public health admissions, $6.1 million from ED presentations), $82.5 million to private health funders, $35 million for MBS item claims and $9.9 for pharmaceutical claims. The high level of costs and service use identified in the first six months post diagnosis are consistent with other research exploring costs for breast cancer [13,27]. After this initial period following diagnosis, costs to all health funders remained relatively consistent over the remaining four and a half years explored in this study.

This study is the first of its kind in Australia to determine long term costs relating to a breast cancer diagnosis, as well as quantifying costs for breast cancer related and non-breast cancer related admissions to both public and private hospitals. Costs relating to non-cancer related admissions took up a greater proportion of average public hospital costs over the entire five-year period for this sample. In contrast, costs relating to cancer admissions in private hospitals were higher than non-cancer related admissions in the first six months post diagnosis. After this initial six months, non-cancer related costs became greater than cancer-related costs for the remaining time. The pattern of results found in this study suggests that private hospitals may have a higher burden of both costs and admissions in the initial period post diagnosis, as females diagnosed with breast cancer seek specialist care and treatment that may only be accessible, or accessible more quickly, through privately funded healthcare. This finding is supported by Australian research which showed that most participants (94%) attended a private hospital to get cancer-related surgery, even if they had a choice between private and public hospitals [28]. Further research is needed to explore factors influencing time to treatment across the public and private sectors in Australia.

For women diagnosed with early-stage breast cancer, surgical procedures (either mastectomy or breast conserving) with radiotherapy are the primary treatment options [29]. If seeking treatment through public hospitals as a public patient, persons with breast cancer are placed on a hospital waiting list for planned surgery, with recommended maximum wait times ranging from within 30 days (urgent); within 90 days (semi-urgent); or non-urgent (no set time frame) [30]. In the private sector, patients can usually access surgery more quickly than in the public sector, especially for semi-urgent or non-urgent cases. Therefore, patients may elect to get their surgery carried out in the private sector to get their surgery performed quicker [28]. The recommendation of seeking initial surgical treatment at a privately funded facility may result in greater admissions and costs for private sector funders in the initial period after diagnosis. Once surgery has been performed in the private sector, patients may then elect to get other continuing treatments as a public patient.

The higher costs incurred by private hospital funders as found in this study may be due to the services that patients access when they visit private healthcare facilities. Research indicates that, due to the mixed public-private healthcare system in Australia, there is potential for ‘cream skimming’, whereby the private sector provides high profit treatment options such as radiotherapy and/or surgery while transferring complex patients back into the public sector [31]. In support of this, research indicates that costly procedures such as immediate breast reconstruction following a mastectomy are more likely to be carried out in private hospitals [32]. This pattern of care be particularly evident through the high average costs identified within private hospitals in the first six months post-diagnosis in this study, where people diagnosed with breast cancer are accessing expensive treatment options in private healthcare facilities to skip the waiting lists that may occur within public hospitals.

The high proportion of costs associated with non-cancer related admissions into both public and private hospitals as shown in this study may be the result of the increased risk of other chronic diseases and general functional decline associated with cancer treatment [33,34]. For example, over a third of long-term survivors (mean time from diagnosis to survey completion of 18 years) of breast cancer reported suffering from arthritis/osteoporosis, a condition which would require regular healthcare post cancer diagnosis. Different treatment options have also been found to have significant health effects in the year following treatment end, with women receiving chemotherapy significantly more likely to report vaginal, musculoskeletal and weight problems than those not receiving chemotherapy [33]. These side effects may contribute to the levels of continued healthcare reported by females with breast cancer within this sample over the remaining four-and-a-half years following the initial six months post diagnosis.

The average out-of-pocket costs and number of MBS services and PBS prescriptions claimed were similar to a study looking at these factors over three years post diagnosis [17]. Of interest here is that the average cost to the individual, and average number of services claimed, remained steady over a five-year period after the initial six months post diagnosis. This suggests that there is a long-term cost burden to the individual relating to a breast cancer diagnosis, as individuals continue to incur similar levels of out-of-pocket costs for items covered by Medicare and under the PBS. While it is not clear if the MBS or PBS items identified in this study were directly related to a cancer diagnosis, the high levels of claims identified in this study may be attributable to the recommendation of involving multidisciplinary teams for continuity of care post breast cancer diagnosis [35]. After initial treatment, the involvement of a multidisciplinary team such as breast cancer nurses, physiotherapists, and psychologists to provide continued healthcare may mean that MBS services are continually being accessed by breast cancer patients over a significant period. While this may be beneficial from a health perspective, the long-term financial costs of this model of care need to be further considered.

By providing costs over a five-year period using linked population-based administrative data, this study provides a unique understanding of the long-term costs of a breast cancer diagnosis borne by different healthcare funders. By using individual record data for an entire state of Australia, a comprehensive assessment of healthcare costs and service use for individuals diagnosed with breast cancer was provided. In capturing all healthcare use and costs for these patients, the results of this study are representative of what is occurring at a population-level within Australia.

Some limitations of this study include the inability to accurately assess costs based on breast cancer stage, and the potential misidentification of hospital admissions as cancer related. Unfortunately, as the QCR does not routinely collect stage of disease at diagnosis, this study was unable to provide accurate information relating to the long-term costs of breast cancer associated with different stages at diagnosis. As such, stage was not explored in this study; however, it is an avenue for further research, as previous studies have identified differences in cancer costs based on breast cancer stage [36]. While providing useful information, the categorization of hospital admissions into cancer and non-cancer related may have provided inaccurate estimates of costs relating to these procedures. It is possible that there were certain hospital admissions within this dataset which would not have been coded as being cancer-related, even though the admission was the side effect of a cancer diagnosis. More refined coding of procedures is required to get a full understanding of the procedures directly related to a breast cancer diagnosis.

## 5. Conclusions

This study was the first of its kind to quantify the long-term costs to the funders of the Queensland healthcare system for public and private hospital admissions, ED presentations and MBS/PBS services for women diagnosed with breast cancer. This paper has implications for health policy and health service planning, with high use of privately funded healthcare in the immediate six months following diagnosis. Health system planning could target these first six months post diagnosis to ensure that newly diagnosed breast cancer patients can access the appropriate services in a timely manner in both public and private hospitals in those key first months of a cancer diagnosis. Planned future studies should have a more detailed investigation of the types of MBS items and pharmaceuticals being claimed by breast cancer patients in the long-term following their diagnosis.

While there is no doubt that the improved survival rates for breast cancer worldwide is an overwhelmingly positive outcome, the increasing rates of both survival and diagnoses for breast cancer means that the long-term costs associated with healthcare for those diagnosed with breast cancer will remain a global issue. To alleviate ongoing costs for breast cancer care, further financial support can be provided to those diagnosed with breast cancer throughout their cancer journey. This could be in the form of insurance payments made to an individual once diagnosed with breast cancer to assist with out-of-pocket costs, and/or more cancer related health services (for example, breast MRI scans) being placed on the MBS so patients can access rebates for these important services. Further, key treatment options such as radiotherapy could be covered by private health insurance, ensuring lower costs for healthcare funders due to a breast cancer diagnosis. Future research should explore the patient perspective on these suggested recommendations, with the aim to reduce costs to all healthcare funders resulting from a breast cancer diagnosis.

## Figures and Tables

**Table 1:** Demographic characteristics at diagnosis of Queensland women with breast cancer diagnosed between 1 July 2011 and 30 June 2013.

	n (%)
Age group	
18–44 years	1247 (23.2)
45–64 years	2184 (40.6)
65 years and above	1952 (36.2)
Indigenous status	
Indigenous women	83 (1.5)
Remoteness	
Metropolitan	2675 (49.9)
Regional	2278 (42.4)
Remote	411 (7.7)
Index of Relative Socio-Economic Disadvantage	
Quintile 1 (most disadvantaged)	361 (6.7)
Quintile 2	225 (4.2)
Quintile 3	907 (16.9)
Quintile 4	2434 (45.4)
Quintile 5 (least disadvantaged)	1437 (26.8)
Breast cancer stage	
Early	2468 (46)
Regional/advanced	2439 (45)
Unknown	476 (9)

**Table 2:** Healthcare costs over five years for Queensland women with breast cancer diagnosed between 1 July 2011 and 30 June 2013.

Healthcare Type	Cost Type	Mean (SD) Cost Per Person	Sum Cost Over 5 Years
Public hospitals	Cancer related costs	$3349 (10,599)	$18,029,931
	Non-cancer related costs	$9174 (25,381)	$49,381,115
	Total cost	$12,523 (29,726)	$67,411,046
Private hospitals	Cancer related costs	$6772 ($11,103)	$36,453,528
	Non-cancer related costs	$8555 ($25,001)	$46,049,002
	Total cost	$15,327 ($29,538)	$82,502,530
Emergency departments		$1132 (2306)	$6,091,598
Medicare Benefits Schedule		$6578 ($7248)	$35,407,985
Pharmaceutical Benefits Scheme		$1836 (1489)	$9,885,472

## Data Availability

The datasets used during the current study are not publicly available due to privacy constraints associated with our ethics approval that explicitly prohibits the sharing of data. The coding used during the current study are not publicly available due to privacy constraints associated with our ethics approval that explicitly prohibits the sharing of code.
